# Fixation, sex, and age: highest risk of revision for uncemented stems in elderly women — data from 66,995 primary total hip arthroplasties in the Norwegian Arthroplasty Register

**DOI:** 10.1080/17453674.2019.1682851

**Published:** 2019-10-30

**Authors:** Håvard Dale, Sjur Børsheim, Torbjørn Berge Kristensen, Anne Marie Fenstad, Jan-Erik Gjertsen, Geir Hallan, Stein Atle Lie, Ove Furnes

**Affiliations:** a The Norwegian Arthroplasty Register, Department of Orthopaedic Surgery, Haukeland University Hospital, Bergen;; b Department of Clinical Medicine, University of Bergen, Bergen;; c Department of Surgery, Voss Hospital, Voss;; d Department of Clinical Dentistry, University of Bergen, Bergen, Norway

## Abstract

Background and purpose — There is no consensus on best method of fixation in hip arthroplasty. We investigated different modes of fixation in primary total hip arthroplasty (THA) and the influence of age and sex, to assess need for a differentiated approach.

Patients and methods — The study was based on data from the Norwegian Arthroplasty Register in the period 2005–2017. Included were all-cemented, all-uncemented, reverse hybrid (uncemented stem and cemented cup), and hybrid (cemented stem and uncemented cup) THA designs that were commonly used, contemporary and well documented, using different causes of revision as endpoints.

Results — From the included 66,995 primary THAs, 2,242 (3.3%) were revised. Compared with all-cemented THAs, all-uncemented had a higher risk of revision due to any cause (RR 1.4; CI 1.2–1.6), mainly due to an increased risk of periprosthetic fracture (RR 5.2; CI 3.2–8.5) and dislocation (RR 2.2; CI 1.5–3.0). Women had considerably higher risk of revision due to periprosthetic fracture after all-uncemented THA (RR 12; CI 6–25), compared with cemented. All-uncemented THAs in women of age 55–75 years (RR 1.3; CI 1.0–1.7) and over 75 years of age (RR 1.8; CI 1.2–2.7), and reverse hybrid THAs in women over the age of 75 (RR 1.5; CI 1.1–1.9) had higher risk of revision compared with cemented. Hybrid THAs (RR 1.0; CI 0.9–1.2) and reverse hybrid THAs (RR 1.0; CI 0.7–1.3) had similar risk of revision due to any cause as cemented THAs.

Interpretation — Uncemented stems (all-uncemented and reverse hybrid THAs) had increased risk of revision in women over 55 years of age, mainly due to periprosthetic fracture and dislocation, and should probably not be used in THA in these patients.

Cemented THAs have been reported to have better overall implant survival than uncemented THAs (Hailer et al. 2010, Mäkelä et al. 2014). Still, there has been a worldwide increase in the use of uncemented THAs, including in elderly patients (Troelsen et al. 2013, Mäkelä et al. 2014). Cemented THAs have been reported to be prone to aseptic loosening, mostly in younger patients, and in the long term, whereas THAs with uncemented components have been prone to revisions due to femoral fractures, dislocations, and infections, often early postoperatively (Pedersen et al. 2014). Differences in prosthesis survival between all-cemented and all-uncemented THAs seem to have evened out during the last decade, and reverse hybrid (uncemented stem and cemented cup) and hybrid fixation (cemented stem and uncemented cup) have shown good results in primary THA (Troelsen et al. 2013, Wyatt et al. 2014, Wangen et al. 2017). In most reports, the outcomes were stratified by age, with results in favor of cemented THAs in the oldest patients. Sex is considered less frequently. There may be need for a more differentiated approach to what mode of fixation would be beneficial for individual patients. One needs to look at all the different reasons for revision in the same cohort. In addition, to eliminate the impact of “poor prostheses” and make the assessment relevant, one should compare the findings in a “best-case” scenario, investigating only commonly used, contemporary, and well- documented prostheses.

We compared prosthesis survival for primary all-cemented, all-uncemented, reverse hybrid (uncemented stem and cemented cup), and hybrid (cemented stem and uncemented cup) THAs relative to sex and age. We assessed the risk of revision for different causes, and assessed whether there were groups of patients in whom certain modes of THA fixation were superior or inferior. 

## Patients and methods

Since its inception in 1987, the Norwegian Arthroplasty Register (NAR) has registered detailed information on primary THAs and THA revisions in Norway. Among the data collected is the patient’s identity, date of operation, indication for primary THA, type of implant, method of fixation, and other surgery-related factors. In addition, information on patient-related factors like sex, age, and comorbidities is registered. The unique identification number of each Norwegian links the primary THA to any subsequent revisions, and the National Population Register, which provides information on death or emigration. The definition of revision is removal or exchange of the whole prosthesis or part(s) of the prosthesis. The surgeon fills in the register form immediately after surgery, and this is mailed and entered electronically at the NAR. The present study is based on validated data from the NAR, with 97% completeness of reporting of primary THAs, 88% reporting of revisions, and 100% coverage of Norwegian hospitals (Furnes et al. 2019).

For this study, we assessed the fixation mode of commonly used, contemporary, and well-documented implants in cases with complete information on patient characteristics. A THA was considered commonly used when both the cup and stem had been used in more than 1,000 THAs, and contemporary when the cup and stem were still in use or used in at least 10 years of the study period. A THA was considered well documented if it had a documented 10-year survival of more than 90%. Whether the THAs were well documented were evaluated through: (1) Results in the NAR, (2) evaluation of the British Orthopaedic Device Evaluation Panel, and lastly (3) results in other arthroplasty registers with sufficient length of follow-up (i.e., Nordic, England and Wales, Australia). 10-year documentation was evaluated at the time of the analyses. Implants with documented poor performance were excluded.

**Figure 1.**
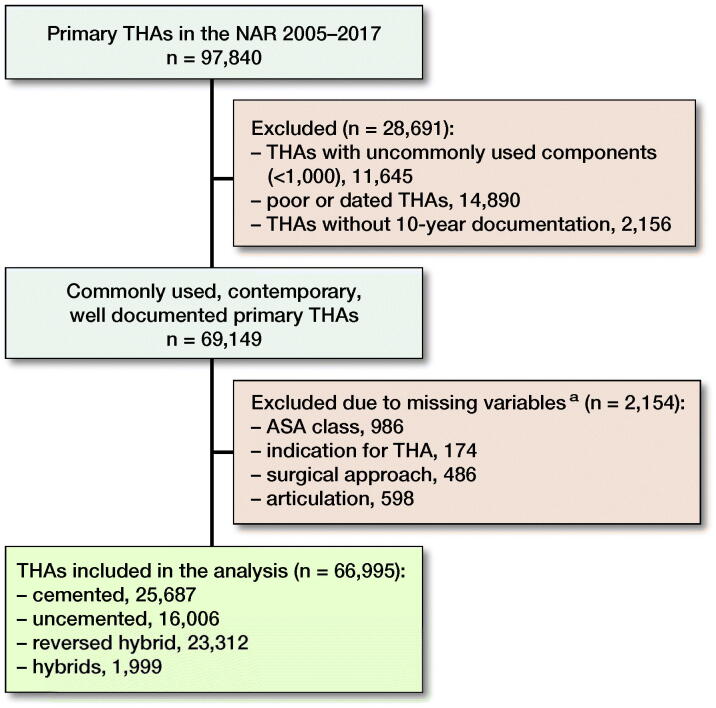
Flowchart of inclusion and exclusion of THAs. a There may be more than 1 missing variable per THA.

Comorbidity according to the ASA classification has been registered in the NAR since 2005. In addition, the use of highly cross-linked polyethylene (XLPE) was established at that time. Therefore, the period of inclusion and observation for the present study was from January 1, 2005 to December 31, 2017. From this time period, the NAR contained data on 97,840 primary THAs. 30,845 THAs were excluded due to infrequent use, poor performance, terminated use, lack of 10-year documentation, or due to missing information on essential variables. In the end, 66,995 primary THAs in 55,935 patients were eligible for analyses (Table 1 and Figure 1).

**Table 1.** Included commonly used, contemporary, and well-documented stems and cups employed in THA in Norway 2005-2017

THA fixation	Stem	Cup
Cemented	Exeter^1^,	Exeter^1^, Elite^4^, IP/SP1^3^,
	Spectron EF^2^,	Contemporary^1^, Marathon^4^,
	Lubinus SP2^3^,	Exeter X3 Rimfit^1^,
	Charnley Modular^4^	Reflection (XLPE)^2^
Uncemented	Corail^4^,	Reflection uncemented^2^,
	Filler^5^,	Trilogy^7^, Igloo^5^, Trident^1^,
	Hactiv^6^	Pinnacle^4^, R3^2^
Reverse hybrid	Corail^4^,	Exeter^1^, Elite^4^, IP/SP1^3^,
	Filler^5^,	Contemporary^1^, Marathon^4^,
	Hactiv^6^	Exeter X3 Rimfit^1^,
		Reflection (XLPE)^2^
Hybrid	Exeter^1^,	Reflection uncemented^2^,
	Spectron EF^2^,	Trilogy^7^, Trident^1^,
	Lubinus SP2^3^,	Pinnacle^4^, R3^2^
	Charnley Modular^4^	

^1^ Stryker, ^2^ Smith & Nephew, ^3^ Waldemar LINK, ^4^ DePuy,

^5^ Biotechni, ^6^ Evolutis, ^7^ Zimmer Biomet.

### Statistics

We performed Kaplan–Meier (KM) survival analyses in addition to adjusted survival analyses by Cox regression models. Time of revision due to any cause or revision due to aseptic loosening, deep infection, periprosthetic fracture, dislocation, or other reasons were the endpoints in the analyses.

All THAs were followed until their first revision, until the date of death or emigration of the patient, or until censoring at December 31, 2017. Patients were censored at time of death or emigration by linkage to the National Population Register.

Adjusted hazard rate ratios, as a measure of relative risk (RR), were estimated for types of fixation, overall, for each sex, and in 3 age groups. In the Cox analyses, we adjusted for sex, age, ASA class, indication for primary THA, surgical approach, articulation, and head size of the prosthesis. Further, we adjusted for year of primary surgery to minimize the effect of time-dependent confounding.

The analyses were performed in accordance with the guidelines for statistical analyses of arthroplasty register data (Ranstam et al. 2011). The proportional hazard assumptions of the Cox survival analyses were not completely fulfilled between the 4 modes of fixation when tested by smoothed Schoenfeld residuals (Figures 3 and 5). This resulted in assessment of the risk of revision 0–1 year, 1–3 years, and 3–10 years postoperatively and in the age groups less than 55, 55–75, and over 75 years.

In earlier register studies from the NAR we found that potential overestimation of incidence of revision through the effect of competing risks (death and revision) is negligible. The competing risk analyses (Fine & Grey) will therefore give similar results to the Cox analyses (Ranstam and Robertsson 2017). Based on this we chose to include results only from KM and Cox analyses. Bilateral THAs are dependent observations, but the influence of bilaterality has been found to have negligible influence on outcome (Lie et al. 2004, Ranstam et al. 2011). Hence, patients with bilateral THAs were included, and considered independent.

95% confidence intervals (CI) were calculated for survival rates and RRs. We used the IBM SPSS 24.0 (IBM Corp, Armonk, NY, USA) and R statistical software (R Centre for Statistical Computing, Vienna, Austria) packages for analyses, and the study was performed in accordance with the STROBE and RECORD statements.

### Ethics, data sharing plan, funding, and potential conflicts of interests

The registration of data and the study was performed confidentially on patient consent and according to Norwegian and EU data protection rules. Data may be accessible upon application to the NAR. The study was fully financed by the NAR, and no conflict of interest is declared.

## Results

65% of the THA patients were women, mean age was 68 years (12–97), and mean ASA class was 2.0. Median follow-up was 4.6 years (interquartile range: 2.1–7.2). The group of cemented THAs had the longest follow-up. In general, patients with uncemented THAs were younger and slightly healthier than those with cemented THAs, with reverse hybrid and hybrid THA patients as intermediate groups (Tables 2 and 3). 66% of the cemented stems were polished taper slip (forced closed) stems.

**Table 2.** Main characteristics of the study population by modes of THA fixation

THA	Cemented	Uncemented	Reverse hybrid	Hybrid
Included THAs	25,678	16,006	23,312	1,999
Included patients	22,537	13,706	20,259	1,689
Revised THAs at 10 years, n (%)	918 (3.6)	535 (3.3)	711 (3.0)	46 (2.3)
Mean follow-up (range), years	5.8 (0–13)	3.9 (0–13)	4.4 (0–13)	3.1 (0–13)
Median follow-up (IQR), years	5.8 (2.9–8.6)	3.7 (1.3–6.3)	4.2 (2.1–6.3)	2.3 (1.1–3.9)
Mean age (range)	72 (25–97)	63 (12–95)	67 (16–97)	68 (21–96)
Mean ASA class	2.1	1.9	2.0	2.0

IQR: interquartile range

**Table 3.** Distribution of patient and surgery related factors by mode of fixation

		Number of THAs	Number revised	Cemented (%)	Uncemented (%)	Reverse hybrid (%)	
Risk factors	n = 66,995	n = 2,210	n = 25,678	n = 16,006	n = 23,312	n = 1,999	Hybrid (%)
Sex							
Male	23,235	989	30	39	36	36	
Female	43,760	1,221	70	61	64	64	
Age							
< 45	2,055	75	0.3	8	3	2	
45–54	5,455	196	2	16	9	10	
55–64	15,707	505	14	31	29	27	
65–74	24,484	784	39	33	37	29	
75–84	16,462	548	40	12	19	27	
≥ 85	2,832	102	7	1	3	6	
ASA class							
1	12,501	368	13	25	20	16	
2	41,924	1,336	62	62	63	68	
3	12,339	495	24	13	17	16	
4	231	11	0.5	0.3	0.2	0.3	
Indication for primary THA							
Osteoarthritis	52,305	1,698	80	72	80	71	
Inflammatory hip disease	1,495	49	2	2	2	1	
Acute hip fracture	1,981	73	4	2	3	1	
Complication after hip fracture	2,978	143	6	3	4	3	
Complication after childhood hip disease	6,169	169	5	17	7	22	
Osteonecrosis of the femoral head	1,683	93	2	3	3	3	
Other diagnosis	384	17	0.6	0.8	0.4	0.3	
Surgical approach							
Anterior	3,390	98	0	9	8	0	
Anterolateral	6,906	240	5	3	23	1	
Lateral	28,469	1,028	54	22	46	8	
Posterolateral	28,230	844	41	66	23	92	
Articulation							
Metal-poly	14,583	626	51	0.2	6	1	
Metal-XLPE	29,827	857	44	31	51	85	
Ceramic-poly	2,505	87	1	2	9	2	
Ceramic-XLPE	16,920	544	4	48	34	10	
Ceramic-ceramic	3,160	96	0	19	0	2	
Head size, mm							
28	31,559	1,219	65	14	53	18	
32	31,557	875	33	71	46	52	
36	3,879	116	2	15	1	30	

**Figure 2.**
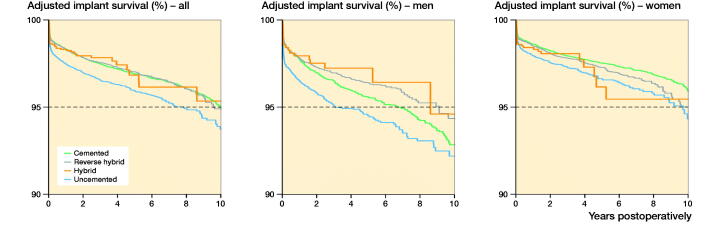
Adjusted implant survival curves with any revision as endpoint, for the 4 types of THA fixation in all THAs, THA in males, and THA in females, adjusted for age, sex (in all THAs only), ASA class, indication for primary THA, surgical approach, articulation, head size of the prosthesis, and year of primary surgery

Among the included 66,995 primary THAs, 2,210 (3.3%) were revised. The 10-year KM survival and adjusted implant survival was 94–95% for all 4 modes of fixation (Figure 2, Table 4, see Supplementary data). However, compared with cemented THAs, uncemented THAs had a 40% higher risk of revision. Reverse hybrid and hybrid THA had a similar risk of revision to cemented (Figure 2, Table 4, see Supplementary data).

**Table 5.** Risks of revision due to different causes, for men and women, for the 4 groups of fixation, adjusted for age, ASA class, indication for primary THA, surgical approach, articulation, head size of prosthesis, and year of primary surgery

		THAs in men			THAs in women		
		THAs	Revisions	Relative risk (CI)	THAs	Revisions	Relative risk (CI)
Aseptic loosening							
Cemented	7,756	83	1	17,922	118	1	
Uncemented	6,296	39	0.9 (0.5–1.6)	9,710	39	0.5 (0.3–0.8)	
Reverse hybrid	8,466	69	0.9 (0.6–1.5)	14,846	99	1.0 (0.7–1.4)	
Hybrid	717	2	0.5 (0.1–2.1)	1,282	1	0.2 (0.0–1.2)	
Infection							
Cemented	7,756	156	1	17,922	193	1	
Uncemented	6,296	106	1.1 (0.8–1.6)	9,710	66	1.3 (0.9–1.9)	
Reverse hybrid	8,466	126	0.7 (0.5–0.9)	14,846	119	0.9 (0.7–1.2)	
Hybrid	717	9	0.8 (0.4–1.6)	1,282	13	1.5 (0.8–2.8)	
Periprosthetic fracture							
Cemented	7,756	33	1	17,922	23	1	
Uncemented	6,296	26	1.8 (0.9–3.6)	9,710	41	12.3 (6.2–24)	
Reverse hybrid	8,466	33	1.4 (0.7–2.6)	14,846	81	9.9 (5.6–18)	
Hybrid	717	0		1,282	4	7.4 (2.3–24)	
Dislocation							
Cemented	7,756	84	1	17,922	156	1	
Uncemented	6,296	71	2.6 (1.6–4.4)	9,710	65	1.8 (1.1–2.8)	
Reverse hybrid	8,466	31	0.6 (0.4–1.0)	14,846	50	0.7 (0.4–1.0)	
Hybrid	717	5	1.1 (0.4–3.0)	1,282	6	1.2 (0.5–2.9)	
Other							
Cemented	7,756	24	1	17,922	48	1	
Uncemented	6,296	39	1.4 (0.6–3.0)	9,710	43	1.2 (0.6–2.2)	
Reverse hybrid	8,466	50	1.5 (0.8–2.9)	14,846	53	0.9 (0.6–1.6)	
Hybrid	717	3	1.6 (0.4–5.9)	1,282	3	1.0 (0.3–3.5)	

### Fixation and sex

Men had a higher risk of revision (RR 1.6; CI 1.4–1.7) than women (Figure 2). The risk of revision after uncemented THA was higher in both men and women, whereas reverse hybrid and hybrid THAs had similar overall revision risks compared with cemented THAs within each sex (Figure 2, Table 4, see Supplementary data).

### Fixation, sex, and age

**Figure 3.**
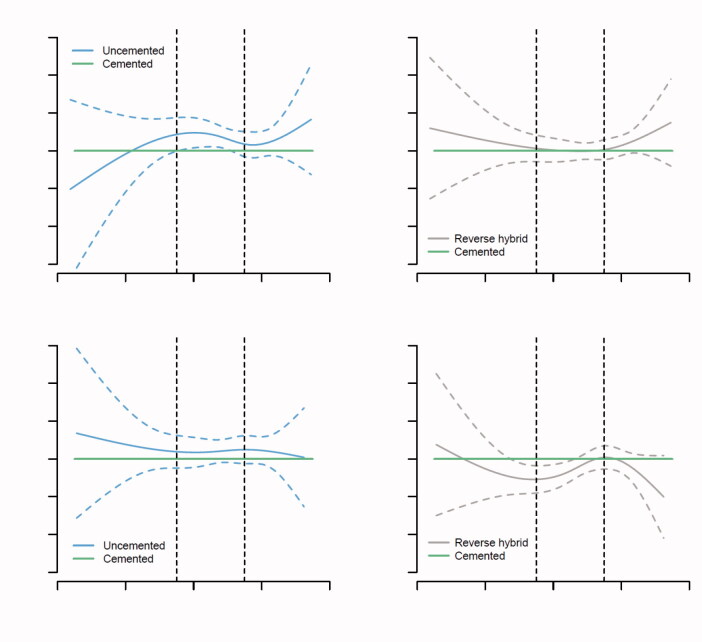
Graphical representation of the relationship between age at primary THA and the log relative risk (RR) for revision due to all causes for uncemented and reverse hybrid compared with cemented THAs, for women and men with 95% confidence intervals. The horizontal green line shows the reference hazard rate ratio (RR = 1) of cemented THAs. The vertical lines indicate 55 and 75 years of age. We adjusted for ASA class, indication for primary THA, surgical approach, articulation, head size of the prosthesis, and year of primary surgery in the analyses. Hybrid THAs are omitted due to low numbers.

In women the risk of revision after uncemented THA, compared with cemented, increased with age (Figure 3, Table 4, see Supplementary data). In addition, the risk of revision after reverse hybrid THA, compared with cemented THA, was increased in women older than 75 years (Figure 3, Table 4, see Supplementary data).

In men, the risk of revision after uncemented THA was increased compared with cemented THAs (Figures 2 and 3, Table 4, see Supplementary data). However, in contrast to women, the results for uncemented and reverse hybrid THAs were similar to cemented THAs in men over 55 years of age (Figures 2 and 3, Table 4, see Supplementary data). Nevertheless, there was a trend of increased risk of revision for uncemented, compared with cemented, THAs for men over 75 years of age (Figures 2 and 3, Table 4, see Supplementary data).

**Figure 4.**
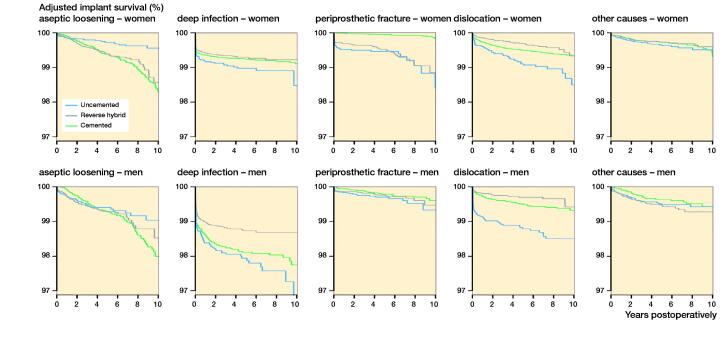
Adjusted implant survival curves for different causes of revision for 3 types of THA fixation in women and men, adjusted for age, ASA class, indication for primary THA, surgical approach, articulation, head size of the prosthesis, and year of primary surgery. Hybrid THAs are omitted due to low numbers.

### Fixation, sex, and causes of revision

Deep infection (1.2 %) was the most common cause of revision, followed by dislocation (0.7%), aseptic loosening (0.7%), periprosthetic fractures (0.4%), and other causes of revision (pain, wear, breakage of components, osteolysis, anisomelia, etc.) (0.4%). 93% of the periprosthetic fractures involved the femur and 7% the acetabulum.

In men, the risk of revision due to infection was slightly lower after reverse hybrid THA, compared with cemented (Table 5, Figure 4). The risk of revision due to dislocation, however, was 2.6-fold increased after uncemented THA, compared with cemented (Table 5, Figure 4).

In women, the risk of revision due to periprosthetic fracture was grossly increased with an uncemented stem (uncemented or reverse hybrid THA) (Table 5, Figure 4). Even women with hybrid THAs had an increased risk of revision due to periprosthetic fracture, but the number of revisions (4) was very low in this group (Table 5). The risk of revision due to aseptic loosening, however, was decreased after uncemented THA in women, compared with cemented THAs.

### Fixation, sex, causes of revision, and time postoperatively

**Figure 5.**
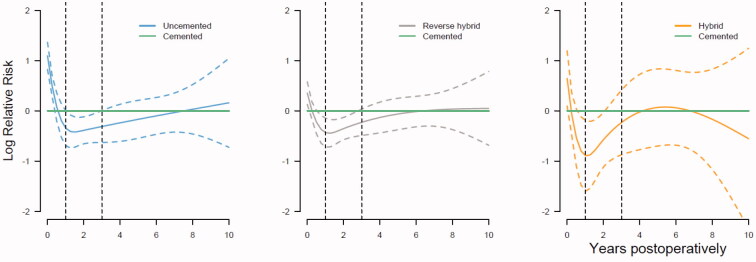
Graphical representation of the relationship between year postoperatively and the log relative risk (RR) for revision due to all causes for uncemented, reverse hybrid, and hybrid THAs, compared with cemented THAs, with 95% confidence intervals. The horizontal green line shows the reference hazard rate ratio (RR = 1) of cemented THAs. The vertical lines indicate 1 and 3 years postoperatively. We adjusted for sex, age, ASA class, indication for primary THA, surgical approach, articulation, head size of the prosthesis, and year of primary surgery in the analyses

Uncemented THAs had an increased risk of revision in the first year postoperatively compared with cemented THAs (Figure 5, Table 6, see Supplementary data). This was mainly due to increased risk of aseptic loosening (or lack of fixation), periprosthetic fracture, and dislocation in both men and women (Table 6, see Supplementary data). During the first year postoperatively, compared with cemented THAs, reverse hybrid THAs also had a higher risk of aseptic loosening in both sexes (Table 6, see Supplementary data). In women, the risk of revision due to periprosthetic fracture after uncemented THA was increased 19-fold in the first year postoperatively, compared with cemented THAs (Table 6, see Supplementary data). In contrast to men, women had an 11 times increased risk of revision due to periprosthetic fracture after reverse hybrid THAs (Table 6, see Supplementary data) in the first year postoperatively.

Between 1 and 3 years, the risk of revision was lower for uncemented, reverse hybrid, and hybrid THAs compared with cemented THAs, in both men and women, mainly due to increased risk of aseptic loosening after cemented THAs (Figure 5, Table 6, see Supplementary data).

From 3 to 10 years postoperatively all 4 modes of fixations had similar overall risk of revision (Figure 5, Table 6, see Supplementary data). However, both men and women had a lower risk of revision due to aseptic loosening after uncemented THA (Table 6, see Supplementary data). Women, in contrast to men, had grossly increased risk of revision due to periprosthetic fracture 3 to 10 years after all THAs involving uncemented components, compared with cemented THAs (Table 6, see Supplementary data).

## Discussion

We found good overall survival for common, contemporary, well-documented primary THAs regardless of fixation method: cemented, uncemented, reverse hybrid, or hybrid fixation. However, uncemented THAs had a slightly higher overall risk of revision compared with cemented THAs. This difference was mainly caused by an increased risk of periprosthetic fracture and dislocation after uncemented THA, in particular when used in elderly women. Reverse hybrid and hybrid THAs had similar overall results to cemented THAs, except for a reverse hybrid in women over the age of 75 years, where the risk of revision was higher.

Traditionally, uncemented THAs have been found, as in our study, to have higher revision rates than cemented THAs (Hailer et al. 2010, Mäkelä et al. 2014, Kandala et al. 2015). Despite this knowledge, there has been a paradoxical increase in the use of uncemented THA (Troelsen et al. 2013, Mäkelä et al. 2014). Recent development of wear-resistant articulating surfaces (i.e., XLPE), together with manufacturers’ marketing skills, may have induced this optimism in uncemented fixation among surgeons (Wechter et al. 2013, Giebaly et al. 2016). At least according to earlier findings from our register, the main problem with earlier generations of uncemented implants was wear and wear-related problems (osteolysis, loosening) (Havelin et al. 2000, 2002, Hallan et al. 2010). There is an increasing bulk of evidence that these issues are less of a problem with modern designs (Broomfield et al. 2017, Devane et al. 2017). Yet another possible reason for the increased usage of uncemented THAs may be inferior results of some commonly used cemented implants (Espehaug et al. 2009, Hallan et al. 2012). We assessed contemporary THAs, in a “best-case” scenario, comprising all patients in a national cohort. We still found inferior results for uncemented THAs, compared with cemented and reverse hybrid THAs. The differences in implant survival were small, but sex and age influenced the results.

Periprosthetic fractures was the revision cause with the most pronounced differences between the sexes. Thus, this is the strongest finding in our study, and has been found by others (Abdel et al. 2016, Wangen et al. 2017, Chatziagorou et al. 2019). Periprosthetic fractures were found to be strongly associated with uncemented and reverse hybrid THAs, and mostly so in women. The fractures were mainly located around the femoral stem. The risk of revision due to periprosthetic fracture was higher in women from the age of 55 and increasing with age. In addition, the risk of revision due to periprosthetic fractures associated with uncemented stems continued to be high up to 10 years postoperatively. This was in contrast to men, where there was only a trend of increased risk of revision due to periprosthetic fracture after 75 years of age, and only early postoperatively. The use of uncemented components in patients with deteriorating bone stock should probably be avoided, since impaction of components may result in fissures due to fragile cortical bone (Piarulli et al. 2013, Sidler-Maier and Waddell 2015, Abdel et al. 2016, Hasegawa et al. 2017, Dammerer et al. 2019). The poorer results after uncemented stems in elderly patients are supported by literature on both THA and hemiarthroplasty (Gjertsen et al. 2012, Mäkelä et al. 2014, Wangen et al. 2017). Womens’ grossly increased risk of periprosthetic fractures with uncemented stems, both early postoperatively and 3–10 years postoperatively, may be due to bone density loss (Alm et al. 2009, Sköldenberg et al. 2014). One of the cemented femoral stems included in our study was the polished taper slipped Exeter™ prosthesis. This implant has a long and successful record of accomplishment. However, polished taper slip (force closed) prostheses have been reported to have an increased risk of periprosthetic fracture (Thien et al. 2014, Palan et al. 2016, Kristensen et al. 2018, Chatziagorou et al. 2019). The Exeter stem (polished taper slip) was used in 66% of the THAs with cemented stems in the present study and inferior outcome with one stem design or brand could potentially affect the whole group. The increased risk of periprosthetic fractures after uncemented THA could therefore have been even more pronounced if other designs of cemented stems were used to a larger degree (Thien et al. 2014). Also on the acetabular side, uncemented components have been associated with periprosthetic fracture (Hasegawa et al. 2017, Dammerer et al. 2019).

The 2nd most common cause of revision was dislocation. Both men and women had an increased risk of revision due to dislocation after uncemented THA, compared with cemented THA, in the first year postoperatively. This was despite the fact that a 28 mm prosthesis head was more common in cemented THAs. In addition, there was higher risk of early aseptic loosening for THAs involving uncemented stems. This may indicate problems with initial stability and orientation of components for uncemented THAs, and in particular stems. Bone stock quality and geometry of the implant may have influenced these findings (Ogino et al. 2008, Finnila et al. 2016). The variation in the orientation of the components, especially the cup, may be larger with uncemented implants (Nishii et al. 2015, Suksathien et al. 2018). Wedge shaped femoral stems, commonly used in Norway, tend to dictate the version of the stem to a large degree (Al-Dirini et al. 2019). Thus, any malpositioning of the cup cannot always be sufficiently adjusted for with the stem. Also, the uncemented stem may subside in the femur more often than cemented ones (Selvaratnam et al. 2015). It may be that these factors lead to suboptimal position of the THA components, and thus to a higher risk of revision due to dislocation. The finding that hybrid and reverse hybrid THAs did not have increased risk of revision due to dislocation may indicate that there is an additive effect on the risk of dislocation when both components are uncemented.

Infection was the most common cause of revision after primary THA. This may partly reflect an increased risk of revision due to infection as reported in other studies (Dale et al. 2012).

Aseptic loosening was the 3rd most common cause of revision. This may confirm that our “best-case” selection included implants with good longevity regarding fixation, as intended. However, it also reflects the relatively short follow-up (median follow-up 4.6 years). A dilemma in evolution of arthroplasty is the conflict of interest between innovation and documentation of longevity. This is also illustrated by the contradiction in the inclusion criteria of the present study: contemporary and well documented. The differences between implants and fixation techniques may only be evident beyond 10–15 years postoperatively. In order to study contemporary THAs, we had a relatively short follow-up. Longer follow-up may change the results, particularly concerning revisions due to aseptic loosening, which is still the most common late cause of revision in studies with long term follow-up (Hailer et al. 2010). The follow-up was also slightly different for the 4 fixation groups as there had been a shift towards increased use of uncemented and reverse hybrid THAs with time. However, we had no indications on improved results for uncemented THAs, compared with cemented, at 10-year follow-up.

### Strengths and limitations

We had the benefit of detailed information on patient- and surgery-related confounders. By way of example, the NAR uses catalogue numbers to identify implants and cements, securing near 100% coding accuracy. Accordingly, we were able to adjust for important differences between the patient groups. Because revisions are relatively rare, it may only be possible to study specific causes of revision in large databases such as national arthroplasty registers. We included a large number of common, contemporary, and well-documented THAs and detailed information on causes of revision and exact survival times. Because the results were based on data from a nationwide THA population, the results should also have good external validity.

Some selection bias and unknown confounding may, however, have affected our results. Patients who received uncemented THAs were in general younger and healthier than those who received cemented THAs. Hospitals with a preference for one type of fixation may have differences in case mix compared with hospitals choosing another fixation for the majority of their patients, differences that were not adjusted for in the analyses. We found that reverse hybrid THAs had a lower risk of revision due to infection, compared with cemented THAs, which could be the result of such bias. However, considering the number of cases, coverage of hospitals, completeness of the data, the strict inclusion criteria, and the fact that we adjusted for several clinically important risk factors in the analyses, we expect the selection bias and unknown confounding to be minor, and the study to be without major systematic errors.

The NAR does not include radiographs nor information on bone stock quality. Assumptions on periprosthetic fractures and fixation relative to elderly women were therefore based on epidemiological and not individual data.

In conclusion, longevity of primary THAs was good for cemented, uncemented, reverse hybrid, and hybrid THAs when common, contemporary, well-documented implants were used. However, uncemented THAs had a higher risk of revision, mainly due to more periprosthetic fractures and dislocations. Uncemented fixation should be considered as best avoided in women aged 55–75 years and avoided in women over the age of 75. The increased risk of revision due to periprosthetic fractures associated with uncemented components in elderly women, as found in our study, has resulted in a quality project in Norway where surgeons are advised to use only cemented stems in women over the age of 75.

## Supplementary Material

Supplemental Material

## Supplementary data

Tables 4 and 6 are available as supplementary data in the online version of this article, http://dx.doi.org/10.1080/17453674. 2019.1682851

## References

[CIT0001] Abdel M P, Watts C D, Houdek M T, Lewallen D G, Berry D J. Epidemiology of periprosthetic fracture of the femur in 32 644 primary total hip arthroplasties: a 40-year experience. Bone Joint J 2016; 98-b (4): 461–7. 27037427 10.1302/0301-620X.98B4.37201

[CIT0002] Al-Dirini R M A, Martelli S, O’Rourke D, Huff D, Zhang J, Clement J G, et al. Virtual trial to evaluate the robustness of cementless femoral stems to patient and surgical variation. J Biomech 2019; 82: 346–56. 30473137 10.1016/j.jbiomech.2018.11.013

[CIT0003] Alm J J, Makinen T J, Lankinen P, Moritz N, Vahlberg T, Aro H T. Female patients with low systemic BMD are prone to bone loss in Gruen zone 7 after cementless total hip arthroplasty. Acta Orthop 2009; 80 (5): 531–7. 19916684 10.3109/17453670903316801 PMC2823339

[CIT0004] Broomfield J A, Malak T T, Thomas G E, Palmer A J, Taylor A, Glyn-Jones S. The relationship between polyethylene wear and periprosthetic osteolysis in total hip arthroplasty at 12 years in a randomized controlled trial cohort. J Arthroplasty 2017; 32 (4): 1186–91. 27998657 10.1016/j.arth.2016.10.037

[CIT0005] Chatziagorou G, Lindahl H, Karrholm J. The design of the cemented stem influences the risk of Vancouver type B fractures, but not of type C: an analysis of 82,837 Lubinus SPII and Exeter Polished stems. Acta Orthop 2019: 1–13. 10.1080/17453674.2019.1574387 PMC6461110 30739553

[CIT0006] Dale H, Fenstad A M, Hallan G, Havelin L I, Furnes O, Overgaard S, et al. Increasing risk of prosthetic joint infection after total hip arthroplasty. Acta Orthop 2012; 83 (5): 449–58. 23083433 10.3109/17453674.2012.733918 PMC3488170

[CIT0007] Dammerer D, Putzer D, Glodny B, Petersen J, Arrich F, Krismer M, Biedermann R. Occult intra-operative periprosthetic fractures of the acetabulum may affect implant survival. Int Orthop 2019; 43(7): 1583–90. 30097730 10.1007/s00264-018-4084-7

[CIT0008] Devane P A, Horne J G, Ashmore A, Mutimer J, Kim W, Stanley J. Highly cross-linked polyethylene reduces wear and revision rates in total hip arthroplasty: a 10-year double-blinded randomized controlled trial. J Bone Joint Surg Am 2017; 99 (20): 1703–14. 29040124 10.2106/JBJS.16.00878

[CIT0009] Espehaug B, Furnes O, Engesaeter L B, Havelin L I. 18 years of results with cemented primary hip prostheses in the Norwegian Arthroplasty Register: concerns about some newer implants. Acta Orthop 2009; 80 (4): 402–12. 19857178 10.3109/17453670903161124 PMC2823190

[CIT0010] Finnila S, Moritz N, Svedstro M E, Alm J J, Aro H T. Increased migration of uncemented acetabular cups in female total hip arthroplasty patients with low systemic bone mineral density. A 2-year RSA and 8-year radiographic follow-up study of 34 patients. Acta Orthop 2016; 87 (1): 48–54. 26569616 10.3109/17453674.2015.1115312 PMC4940591

[CIT0011] Furnes O, Hallan G, Gjertsen J E, Visnes H, Gundersen T, Fenstad A M, et al. The Norwegian Arthroplasty Register, Annual Report; 2019. http://nrlweb.ihelse.net/eng/Rapporter/Report2019_english.pdf.

[CIT0012] Giebaly D E, Twaij H, Ibrahim M, Haddad F S. Cementless hip implants: an expanding choice. Hip Int 2016; 26 (5): 413–23. 27689504 10.5301/hipint.5000423

[CIT0013] Gjertsen J E, Lie S A, Vinje T, Engesaeter L B, Hallan G, Matre K, et al. More re-operations after uncemented than cemented hemiarthroplasty used in the treatment of displaced fractures of the femoral neck: an observational study of 11,116 hemiarthroplasties from a national register. J Bone Joint Surg Br 2012; 94 (8): 1113–19. 22844055 10.1302/0301-620X.94B8.29155

[CIT0014] Hailer N P, Garellick G, Kärrholm J. Uncemented and cemented primary total hip arthroplasty in the Swedish Hip Arthroplasty Register. Acta Orthop 2010; 81 (1): 34–41. 20180715 10.3109/17453671003685400 PMC2856202

[CIT0015] Hallan G, Dybvik E, Furnes O, Havelin L I. Metal-backed acetabular components with conventional polyethylene: a review of 9113 primary components with a follow-up of 20 years. J Bone Joint Surg Br 2010; 92 (2): 196–201. 20130308 10.1302/0301-620X.92B2.22179

[CIT0016] Hallan G, Espehaug B, Furnes O, Wangen H, Hol P J, Ellison P, et al. Is there still a place for the cemented titanium femoral stem? 10,108 cases from the Norwegian Arthroplasty Register. Acta Orthop 2012; 83 (1): 1–6. 22206445 10.3109/17453674.2011.645194 PMC3278649

[CIT0017] Hasegawa K, Kabata T, Kajino Y, Inoue D, Tsuchiya H. Periprosthetic occult fractures of the acetabulum occur frequently during primary THA. Clin Orthop Relat Res 2017; 475 (2): 484–94. 27800574 10.1007/s11999-016-5138-z PMC5213950

[CIT0018] Havelin L I, Engesaeter L B, Espehaug B, Furnes O, Lie S A, Vollset S E. The Norwegian Arthroplasty Register: 11 years and 73,000 arthroplasties. Acta Orthop Scand 2000; 71 (4): 337–53. 11028881 10.1080/000164700317393321

[CIT0019] Havelin L I, Espehaug B, Engesaeter L B. The performance of two hydroxyapatite-coated acetabular cups compared with Charnley cups: from the Norwegian Arthroplasty Register. J Bone Joint Surg Br 2002; 84 (6): 839–45. 12211674 10.1302/0301-620x.84b6.12492

[CIT0020] Kandala N B, Connock M, Pulikottil-Jacob R, Sutcliffe P, Crowther M J, Grove A, et al. Setting benchmark revision rates for total hip replacement: analysis of registry evidence. BMJ 2015; 350: h756. 10.1136/bmj.h756 25752749

[CIT0021] Kristensen T B, Dybvik E, Furnes O, Engesaeter L B, Gjertsen J E. More reoperations for periprosthetic fracture after cemented hemiarthroplasty with polished taper-slip stems than after anatomical and straight stems in the treatment of hip fractures. Bone Joint J 2018; 100-b (12): 1565–71. 30499310 10.1302/0301-620X.100B12.BJJ-2018-0262.R1

[CIT0022] Lie S A, Engesaeter L B, Havelin L I, Gjessing H K, Vollset S E. Dependency issues in survival analyses of 55,782 primary hip replacements from 47,355 patients. StatMed 2004; 23 (20): 3227–40. 10.1002/sim.1905 15449328

[CIT0023] Mäkelä K T, Matilainen M, Pulkkinen P, Fenstad A M, Havelin L, Engesaeter L, et al. Failure rate of cemented and uncemented total hip replacements: register study of combined Nordic database of four nations. BMJ 2014; 348: f7592. 10.1136/bmj.f7592 24418635

[CIT0024] Nishii T, Sakai T, Takao M, Sugano N. Fluctuation of cup orientation during press-fit insertion: a possible cause of malpositioning. J Arthroplasty 2015; 30 (10): 1847–51. 25971534 10.1016/j.arth.2015.04.037

[CIT0025] Ogino D, Kawaji H, Konttinen L, Lehto M, Rantanen P, Malmivaara A, et al. Total hip replacement in patients eighty years of age and older. J Bone Joint Surg Am 2008; 90 (9): 1884–90. 18762648 10.2106/JBJS.G.00147

[CIT0026] Palan J, Smith M C, Gregg P, Mellon S, Kulkarni A, Tucker K, et al. The influence of cemented femoral stem choice on the incidence of revision for periprosthetic fracture after primary total hip arthroplasty: an analysis of national joint registry data. Bone Joint J 2016; 98-b (10): 1347–54. 27694588 10.1302/0301-620X.98B10.36534

[CIT0027] Pedersen A B, Mehnert F, Havelin L I, Furnes O, Herberts P, Kärrholm J, et al. Association between fixation technique and revision risk in total hip arthroplasty patients younger than 55 years of age: results from the Nordic Arthroplasty Register Association. Osteoarthritis Cartilage 2014; 22 (5): 659–67. 24631923 10.1016/j.joca.2014.03.005

[CIT0028] Piarulli G, Rossi A, Zatti G. Osseointegration in the elderly. Aging Clin Exp Res 2013; 25 Suppl 1: S59-60. 24046030 10.1007/s40520-013-0103-0

[CIT0029] Ranstam J, Kärrholm J, Pulkkinen P, Mäkelä K, Espehaug B, Pedersen A B, et al. Statistical analysis of arthroplasty data, II: Guidelines. Acta Orthop 2011; 82 (3): 258–67. 21619500 10.3109/17453674.2011.588863 PMC3235302

[CIT0030] Ranstam J, Robertsson O. The Cox model is better than the Fine and Gray model when estimating relative revision risks from arthroplasty register data. Acta Orthop 2017; 88 (6): 578–80. 28771059 10.1080/17453674.2017.1361130 PMC5694799

[CIT0031] Selvaratnam V, Shetty V, Sahni V. Subsidence in collarless Corail hip replacement. Open Orthop J 2015; 9: 194–7. 26069515 10.2174/1874325001509010194 PMC4460213

[CIT0032] Sidler-Maier C C, Waddell J P. Incidence and predisposing factors of periprosthetic proximal femoral fractures: a literature review. Int Orthop 2015; 39 (9): 1673–82. 25813458 10.1007/s00264-015-2721-y

[CIT0033] Sköldenberg O G, Sjoo H, Kelly-Pettersson P, Boden H, Eisler T, Stark A, et al. Good stability but high periprosthetic bone mineral loss and late-occurring periprosthetic fractures with use of uncemented tapered femoral stems in patients with a femoral neck fracture. Acta Orthop 2014; 85 (4): 396–402. 24954490 10.3109/17453674.2014.931195 PMC4105771

[CIT0034] Suksathien Y, Sueajui J, Piyapromdee U. Deviation of cup alignment from target angle during press-fit insertion. Comput Assist Surg (Abingdon) 2018; 23 (1): 53–6. 30369268 10.1080/24699322.2018.1533040

[CIT0035] Thien T M, Chatziagorou G, Garellick G, Furnes O, Havelin L I, Mäkelä K, et al. Periprosthetic femoral fracture within two years after total hip replacement: analysis of 437,629 operations in the Nordic arthroplasty register association database. J Bone Joint Surg Am 2014; 96 (19): e167. 25274795 10.2106/JBJS.M.00643

[CIT0036] Troelsen A, Malchau E, Sillesen N, Malchau H. A review of current fixation use and registry outcomes in total hip arthroplasty: the uncemented paradox. Clin Orthop Relat Res 2013; 471 (7): 2052–9. 23539124 10.1007/s11999-013-2941-7 PMC3676623

[CIT0037] Wangen H, Havelin L I, Fenstad A M, Hallan G, Furnes O, Pedersen A B, Overgaard S, Kärrholm J, Garellick G, Mäkelä K, Eskelinen A, Nordsletten L. Reverse hybrid total hip arthroplasty. Acta Orthop 2017; 88 (3): 248–54. 28095724 10.1080/17453674.2016.1278345 PMC5434590

[CIT0038] Wechter J, Comfort T K, Tatman P, Mehle S, Gioe T J. Improved survival of uncemented versus cemented femoral stems in patients aged < 70 years in a community total joint registry. Clin Orthop Relat Res 2013; 471 (11): 3588–95. 23873609 10.1007/s11999-013-3182-5 PMC3792261

[CIT0039] Wyatt M, Hooper G, Frampton C, Rothwell A. Survival outcomes of cemented compared to uncemented stems in primary total hip replacement. World J Orthop 2014; 5 (5): 591–6. 25405087 10.5312/wjo.v5.i5.591 PMC4133466

