# Supplementary material for: Fixation, sex, and age: highest risk of revision for uncemented stems in elderly women — data from 66,995 primary total hip arthroplasties in the Norwegian Arthroplasty Register
Source: Acta Orthop. 2019 Oct 30;91(1):33–41. doi: 10.1080/17453674.2019.1682851 (PMC7006785; doi:10.1080/17453674.2019.1682851)
Supplement: Supplemental Material [file IORT_A_1682851_SM7731.pdf]

Supplementary data

Table 4. Overall, sex, and age group-wise risks of revision for the 4 groups of fixation, adjusted for sex (in all THAs only), age, ASA class, indication for primary THA, surgical approach, articulation, head size of the prosthesis and year of primary surgery, in addition to estimated 10-year Kaplan–Meier and adjusted Cox implant survival

Factor	THAs	Revised within 10 years	Relative risk (CI)	10-year KM survival (%)	Adjusted 10-year survival (%)	Censored before 10 years	At risk at 10 years
**All THAs**							
Cemented	25,678	918	1	95 (94–95)	95 (94–96)	4,928	3,390
Uncemented	16,006	535	1.4 (1.2–1.6)	95 (94–95)	94 (93–95)	916	884
Reverse hybrid	23,312	711	1.0 (0.9–1.1)	95(94–96)	95 (94–96)	1,865	950
Hybrid	1,999	46	1.0 (0.7–1.3)	96 (94–98)	95 (93–98)	78	85
**THA in men**							
Cemented	7,756	380	1	93 (92–94)	93 (92–94)	1,780	948
Uncemented	6,296	281	1.3 (1.0–1.6)	93 (92–94)	92 (91–94)	421	355
Reverse hybrid	8,466	309	0.8 (0.7–1.0)	94 (93–95)	94(93–96)	810	356
Hybrid	717	19	0.8 (0.5–1.2)	95 (92–99)	95 (91–99)	29	34
< 55 years							
Cemented	259	13	1	93 (89–97)	93 (54–100)	24	71
Uncemented	1,756	85	1.4 (0.6–3.4)	92 (90–95)	93 (54–100)	47	133
Reverse hybrid	1,270	46	0.8 (0.3–1.8)	94 (91–96)	95 (68–100)	54	98
Hybrid	119	0				4	22
55–75 years							
Cemented	4,368	209	1	93 (92–94)	93 (91–94)	668	678
Uncemented	3,859	161	1.2 (0.9–1.6)	94 (93–95)	94 (92–95)	213	199
Reverse hybrid	5,623	192	0.8 (0.6–1.0)	95 (94–96)	95 (94–96)	394	247
Hybrid	438	14	0.9 (0.5–1.6)	92 (85–99)	91 (84–99)	12	11
> 75 years							
Cemented	3,129	165	1	93 (92–94)	94 (92–95)	1,088	199
Uncemented	681	40	1.6 (1.0–2.5)	92 (89–95)	91 (87–95)	161	23
Reverse hybrid	1,573	72	0.9 (0.7–1.3)	95 (94–96)	95 (94–97)	362	11
Hybrid	160	5	1.0 (0.4–2.6)	97 (94–100)	96 (92–100)	13	1
**THA in women**							
Cemented	17,922	538	1	96 (95–96)	96 (95–97)	3,148	2,442
Uncemented	9,710	254	1.4 (1.1–1.7)	96 (95–96)	94 (93–96)	495	529
Reverse hybrid	14,846	402	1.1 (1.0–1.3)	95 (95–96)	95 (94–96)	1,055	594
Hybrid	1,282	27	1.2 (0.8–1.8)	96 (94–98)	96 (93–98)	49	51
< 55 years							
Cemented	336	18	1	93 (89–96)	94 (86–100)	24	86
Uncemented	2,014	58	1.2 (0.5–2.6)	95 (93–97)	94 (88–100)	38	162
Reverse hybrid	1,627	51	0.9 (0.4–1.9)	94 (92–96)	94 (86–100)	35	150
Hybrid	129	4	1.1 (0.3–3.6)	96 (92–100)	96 (89–100)	4	24
55–75 years							
Cemented	9,219	317	1	95 (94–96)	95 (94–97)	918	1,598
Uncemented	6,274	155	1.3 (1.0–1.7)	96 (96–97)	94 (92–96)	236	313
Reverse hybrid	9,750	248	1.0 (0.8–1.3)	96 (95–97)	96 (95–97)	486	417
Hybrid	660	16	1.4 (0.8–2.4)	95 (92–99)	94 (90–99)	13	25
> 75 years							
Cemented	8,367	217	1	97 (96–97)	97 (96–98)	2,206	758
Uncemented	1,422	44	1.8 (1.2–2.7)	97 (96–98)	96 (94–97)	221	54
Reverse hybrid	3,469	105	1.5 (1.1–1.9)	94 (90–98)	94 (89–98)	534	27
Hybrid	493	7	0.9 (0.4–1.9)	99 (98–100)	99 (97–100)	32	2

The implant survival may be overestimated since not all THAs have a full 10 years of follow-up.

Table 6. Revision risks relative to time after primary THA, for all THAs, and THAs in each sex separately, adjusted for sex (in All THAs only), age, ASA class, indication for primary THA, surgical approach, articulation, head size of prosthesis and year of primary surgery

All THAs	THAs	0–1 year postoperatively Revisions	Relative risk (CI)	1–3 years postoperatively Revisions	Relative risk (CI)	3–10 years postoperatively Revisions	Relative risk (CI)
**All revisions**							
Cemented	25,678	460	1	199	1	259	1
Uncemented	16,006	362	2.0 (1.7–2.5)	90	0.6 (0.4–0.9)	83	0.8 (0.5–1.2)
Reverse hybrid	23,312	428	1.2 (1.0–1.4)	146	0.6 (0.5–0.8)	137	1.0 (0.7–1.3)
Hybrid	1,999	33	1.2 (0.8–1.7)	6	0.4 (0.2–1.0)	7	0.9 (0.4–2.0)
**Men**							
All revisions							
Cemented	7,756	183	1	85	1	112	1
Uncemented	6,296	193	1.9 (1.5–2.6)	52	0.7 (0.4–1.2)	36	0.7 (0.4–1.2)
Reverse hybrid	8,466	197	1.0 (0.8–1.3)	62	0.5 (0.3–0.8)	50	0.7 (0.4–1.1)
Hybrid	717	14	1.1 (0.6–1.9)	3	0.4 (0.1–1.4)	2	0.4 (0.1–1.6)
Aseptic loosening							
Cemented	7,756	7	1	24	1	52	1
Uncemented	6,296	19	7.2 (2.2–24)	12	0.6 (0.2–1.8)	8	0.2 (0.0–0.7)
Reverse hybrid	8,466	28	5.0 (1.8–14)	23	0.6 (0.3–1.4)	18	0.5 (0.3–1.1)
Hybrid	717	1	4.3 (0.5–40)	0		1	0.3 (0.0–2.5)
Infection							
Cemented	7,756	119	1	25	1	12	1
Uncemented	6,296	87	1.2 (0.8–1.7	10	0.7 (0.3–2.1)	9	2.2 (0.5–9.6)
Reverse hybrid	8,466	108	0.7 (0.5–1.0)	13	0.4 (0.2–1.0)	5	0.5 (0.1–2.1)
Hybrid	717	9	0.9 (0.4–1.8)	0		0	
Periprosthetic fracture							
Cemented	7,756	9	1	8	1	16	1
Uncemented	6,296	15	3.3 (1.1–9.6)	5	0.8 (0.2–3.7)	6	1.2 (0.3–4.1)
Reverse hybrid	8,466	20	2.2 (0.8–5.8)	4	0.6 (0.1–2.5)	9	1.2 (0.4–3.7)
Hybrid	717	0		0		0	
Dislocation							
Cemented	7,756	43	1	18	1	23	1
Uncemented	6,296	53	5.3 (2.8–10)	9	0.5 (0.1–2.0)	9	0.9 (0.2–3.2)
Reverse hybrid	8,466	20	0.8 (0.4–1.5)	5	0.3 (0.1–1.2)	6	0.4 (0.1–1.4)
Hybrid	717	2	1.2 (0.3–5.5)	2	0.9 (0.2–4.6)	1	0.5 (0.1–4.9)
Other							
Cemented	7,756	5	1	10	1	9	1
Uncemented	6,296	19	3.8 (1.1–14)	16	0.9 (0.3–3.0)	4	
Reverse hybrid	8,466	21	2.9 (0.9–9.1)	17	0.7 (0.3–2.1)	12	1.7 (0.5–5.6)
Hybrid	717	2	4.5 (0.7–27)	1	1.2 (0.1–11)	0	
**Women**							
All revisions							
Cemented	17,922	277	1	114	1	147	1
Uncemented	9,710	169	2.1 (1.7–2.8)	38	0.5 (0.3–0.9)	47	0.9 (0.5–1.5)
Reverse hybrid	14,846	231	1.3 (1.0–1.6)	84	0.7 (0.5–1.0)	87	1.2 (0.8–1.7)
Hybrid	1,282	19	1.2 (0.7–2.0)	3	0.5 (0.2–1.7)	5	1.7 (0.6–4.3)
Aseptic loosening							
Cemented	17,922	14	1	36	1	68	1
Uncemented	9,710	23	4.4 (1.7–11)	6	0.1 (0.0–0.4)	10	0.1 (0.0–0.4)
Reverse hybrid	14,846	29	3.2 (1.5–7.2)	38	0.7 (0.4–1.2)	32	0.6 (0.3–1.1)
Hybrid	1,282	0		0		1	0.2 (0.1–1.8)
Infection							
Cemented	17,922	162	1	16	1	15	1
Uncemented	9,710	56	1.5 (1.0–2.1)	5	1.0 (0.3–3.7)	5	0.5 (0.1–1.8)
Reverse hybrid	14,846	102	1.0 (0.7–1.3)	11	0.5 (0.2–1.5)	6	0.5 (0.1–2.3)
Hybrid	1,282	13	1.6 (0.8–3.0)	0		0	
Periprosthetic fracture							
Cemented	17,922	7	1	4	1	12	1
Uncemented	9,710	33	19 (7.3–52)	1	1.2 (0.1–15)	7	4.3 (1.0–19)
Reverse hybrid	14,846	52	11 (4.3–27)	6	4.8 (0.9–26)	23	12 (5.0–27)
Hybrid	1,282	2	4.6 (0.8–25)	0		2	41 (7.6–224)
Dislocation							
Cemented	17,922	78	1	41	1	37	1
Uncemented	9,710	34	2.1 (1.1–3.7)	14	1.2 (0.4–3.3)	17	1.8 (0.7–4.6)
Reverse hybrid	14,846	23	0.7 (0.4–1.2)	10	0.6 (0.2–1.3)	17	0.8 (0.4–1.9)
Hybrid	1,282	3	1.0 (0.3–3.5)	2	1.6 (0.3–7.7)	1	1.1 (0.1–8.6)
Other							
Cemented	17,922	16	1	17	1	15	1
Uncemented	9,710	23	1.9 (0.8–4.3)	12	0.6 (0.2–1.8)	8	1.3 (0.3–6.9)
Reverse hybrid	14,846	25	1.1 (0.5–2.4)	19	0.6 (0.2–1.4)	9	1.2 (0.4–4.0)
Hybrid	1,282	1	0.7 (0.1–5.5)	1	0.8 (0.1–6.8)	1	2.3 (0.2–24)
